# Notes on the Chinese species of *Dianous* group I (Coleoptera, Staphylinidae)

**DOI:** 10.3897/zookeys.342.5842

**Published:** 2013-10-14

**Authors:** Liang Tang, Li-Zhen Li

**Affiliations:** 1 Department of Biology, Shanghai Normal University, 100 Guilin Road, 1st Educational Building 323 Room, Shanghai, 200234 P. R. China

**Keywords:** Coleoptera, Staphylinidae, *Dianous*, new synonym, new records

## Abstract

*Dianous zhujianqingi* Tang & Li, 2011 **syn. n.** is synonymised with *Dianous cyaneovirens* (Cameron, 1930). Additional records of *Dianous yao* Rougemont, 1981, *Dianous haraldi* Puthz, 2000 and *Dianous huanghaoi* Tang & Li, 2011 are provided.

## Introduction

The Chinese species of *Dianous* group I were reviewed by Tang and Li in 2011. This group includes nine species: *Dianous yao* Rougemont, 1981, *Dianous tonkinensis* (Puthz), 1968, *Dianous limitaneus* Puthz, 2001, *Dianous viriditinctus* (Champion), 1920, *Dianous fengtingae* Tang & Li, 2010, *Dianous zhujianqingi* Tang & Li, 2010, *Dianous huanghaoi* Tang & Li, 2010, *Dianous shan* Rougemont, 1981 and *Dianous viridicupreus* Rougemont, 1985. Subsequently, more material was received. A study of this material yielded new locality records and a new synonymy.

## Material and methods

For examination of the male genitalia, the last three abdominal segments were detached from the body after softening in hot water. The aedeagus and other dissected parts were mounted in Euparal (Chroma Gesellschaft Schmidt, Koengen, Germany) on plastic slides. Photos of sexual characters were taken with a Canon G7 attached to an Olympus SZX 16 stereoscope; habitus photos were taken with a Canon macro photo lens MP-E 65 mm attached to a Canon EOS40D camera.

The type specimens treated in this study are deposited in the following public and private collections:

cSme private collection Aleš Smetana, Ottawa, Canada

NHMW Naturhistorisches Museum Wien, Austria (Harald Schillhammer)

SNUC Department of Biology, Shanghai Normal University, P. R. China (Li-Zhen Li)

## Taxonomy

### Dianous cyaneovirens

(Cameron, 1930)

http://species-id.net/wiki/Dianous_cyaneovirens

Figs 1 , 2 , 6

Stenus cyaneovirens Cameron, 1930: 335
Dianous cyaneovirens ; Puthz 1981: 106; Rougemont 1985: 131; Rougemont 1987: 49.
Dianous zhujianqingi Tang & Li, 2011: 73. syn. n.

#### Material examined.

**China: Jiangxi, Guizhou:** Type series of *Dianous zhujianqingi* (SNUC); **Guangxi:** 1♂, Jinxiu County, 16 km, 900m, 29.VII.2011, Peng Zhong leg.; 1♀, Jinxiu County, Shengtangshan, 800–1100m, 28.VII.2011, Peng Zhong leg. (SNUC); **Yunnan**: 6♂♂14♀♀, Baoshan City, Baihualing, 1100-1350m, 25°16'N, 98°47'E, 22.IV. 2013, Song, Dai & Peng leg. (SNUC); **Myanmar:** 1♂1♀, Kachin State, ca. 12 km S Putao, W Mularshidi Vill., 500–550m, 27°14.98'N, 97°24.40'E, 2.VI.1999, Schillhammer & Schuh leg. (NHMW); **Nepal:** 1♂2♀♀, Khandbari Dis., Arun Valley at Num main bridge, 1000m, 21.IV.1984, Smetana & Löbl leg. (cSme); 1♂, Khandbari Dis., Induwa Khola Valley, 2000m, 15.IV.84, Smetana & Löbl leg. (cSme)

#### Distribution.

China (Jiangxi, Guizhou, Guangxi, Yunnan), Myanmar, Nepal.

#### Remarks.

The species is reported from the Chinese provinces Yunnan and Guangxi and from Myanmar for the first time. According to the original description of *Dianous zhujianqingi*, the main differences between this species and *Dianous cyaneovirens* are the different coloration and different lengths of the apical portion of the median lobe. Additionally, there was a huge distributional gap between Nepal and East China. However, with more material examined, this distribution gap is filled. The coloration of the species is found to be greatly variable: in the Nepalese populations, the metallic tint of the species varies from golden green to blackish blue, while it is golden green or blue in specimens from Myanmar and China (Guangxi and Yunnan). In the type series of *Dianous zhujianqingi*, all 39 specimens have a faint plumb-coloured tint, except for two specimens from Jiangxi which have a strong brassy tint. The length of the apical portion of the median lobe is also variable: the length is intermediate in specimens from Myanmar and China (Yunnan and Guangxi). For these reasons, *Dianous zhujianqingi* is synonymised with *Dianous cyaneovirens*.

**Figures 1–5.**
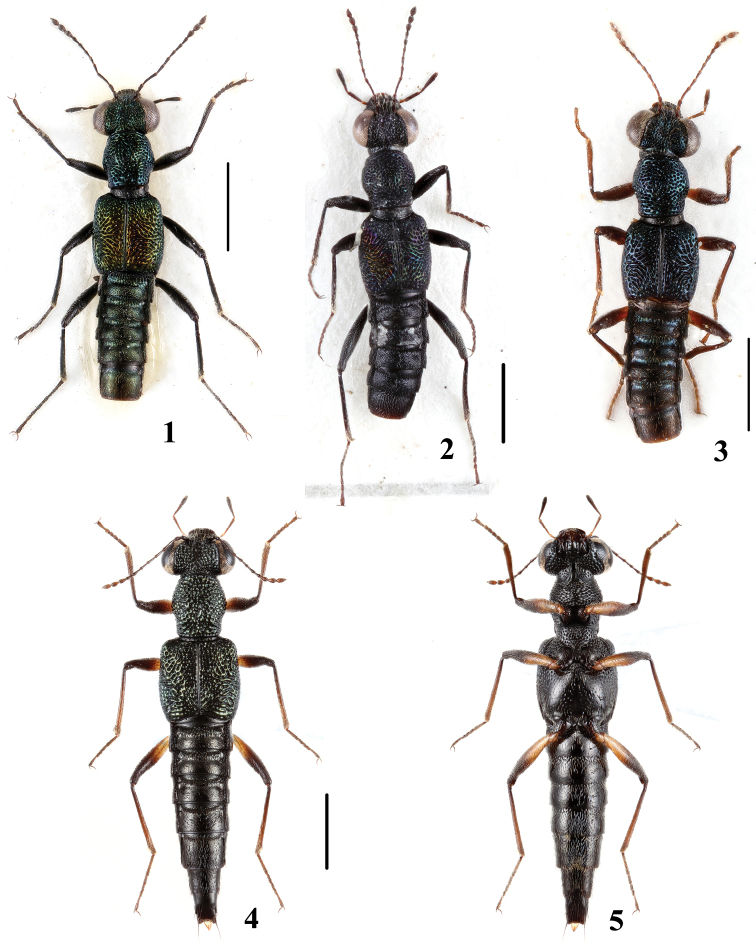
Habitus of *Dianous*. **1** *Dianous cyaneovirens* (Myanmar) **2** *Dianous cyaneovirens* (Nepal) **3** D. *haraldi* **4, 5** *Dianous yao*. Scales = 1 mm.

**Figures 6–9.**
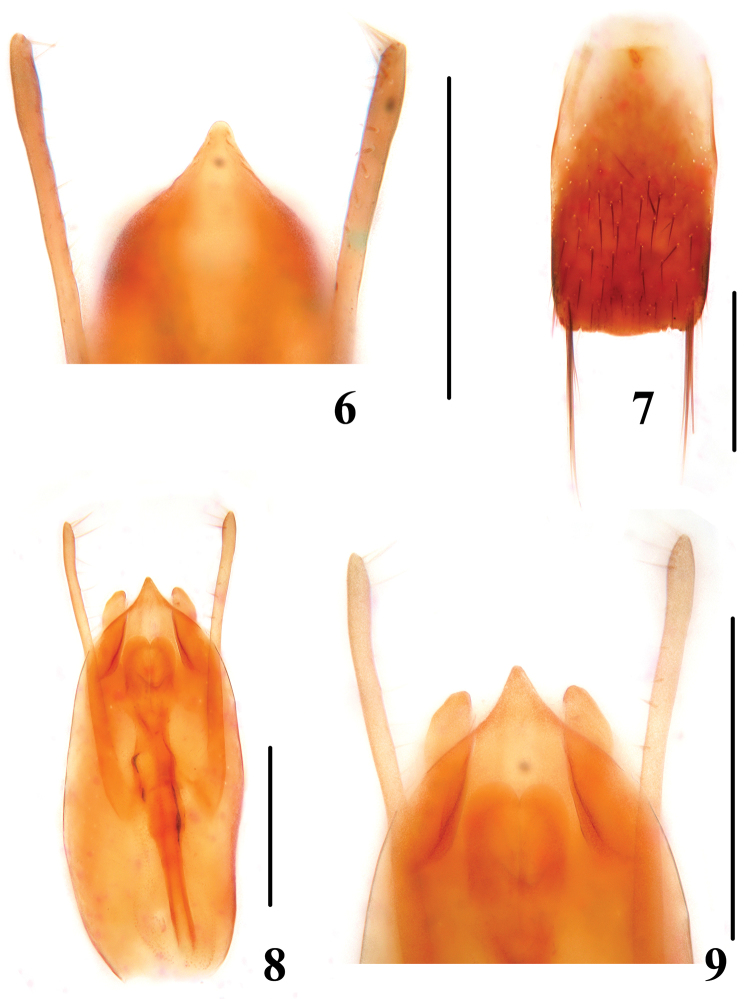
**6** apical portion of median lobe of *Dianous cyaneovirens* (Myanmar) **7–9** *Dianous haraldi*: **7** male sternite IX **8** aedeagus **9** apical portion of median lobe. Scales = 0.25 mm.

### Dianous haraldi

Puthz, 2000

http://species-id.net/wiki/Dianous_haraldi

Figs 3 , 7–9

Dianous haraldi Puthz, 2000: 432.

#### Material examined.

**China: Yunnan**: 1♂, Xishuangbanna, ca. 6km NW Mengla, 700m, 8.VIII.1999, Jäch et al. leg. (CWBS 356) (NHMW)

#### Distribution.

China (Yunnan), Laos.

#### Remarks.

The species is a new record for China

To accommodate *Dianous haraldi*, the recently published key to the Chinese species of *Dianous* group I (Tang, Li and Cao 2012) is modified at couplet 6 as follows:

**Table:** 

6	Forebody black with plumb-coloured lustre, sometimes elytra with brassy reflection; femora unicolored	7
–	Forebody distinctly metallic blue; femora bicolored	7a
7a	Punctation of elytra more confluent; posterior margin of male sternite VII with indistinct median emargination. Habitus (Fig. 3), aedeagus (Fig. 8, 9)	*Dianous haraldi*
–	Punctation of elytra less confluent; posterior margin of male sternite VII with deep median emargination	8

### Dianous huanghaoi

Tang & Li, 2011

http://species-id.net/wiki/Dianous_huanghaoi

Dianous huanghaoi Tang & Li, 2011: 75.

#### Material examined.

**China: Yunnan**: 1♂, Binchuan County, Jizushan, 2400m, 18.VII.2010, Tang leg. (SNUC)

#### Distribution.

China (Yunnan).

#### Remarks.

Previously, the species was only known from Yulongshan and Hutiaoxia in Yunnan.

### Dianous yao

Rougemont, 1981

http://species-id.net/wiki/Dianous_yao

Figs 4 , 5 , 10–19

Dianous yao Rougemont, 1981: 330.

#### Material examined.

**China: Yunnan**: 35♂♂31♀♀, Baoshan City, Baihualing, 1100-1350m, 25°16'N, 98°47'E, 22.IV. 2013, Song, Dai & Peng leg. (SNUC)

#### Distribution.

China (Guizhou, Yunnan); Myanmar, Thailand.

#### Remarks.

The species is new to the Chinese province Yunnan.

**Figures 10–19.**
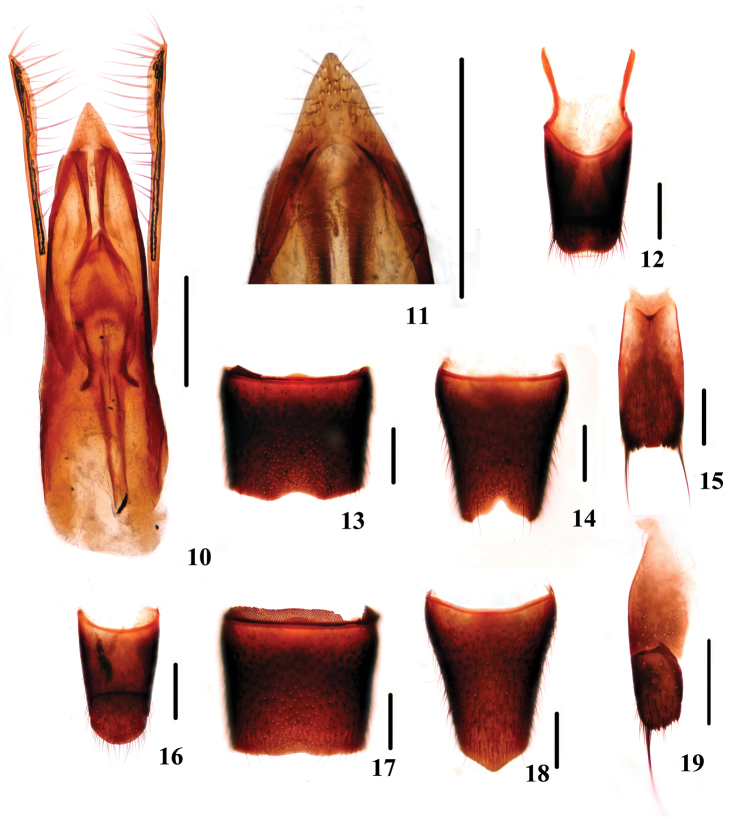
Sexual characters of *Dianous yao*. **10** aedeagus **11** apical portion of median lobe **12** tergites IX uand X **13** male sternite VII **14** male sternite VIII **15** male sternite IX **16** female tergites IX and X **17** female sternite VII **18** female sternite VIII **15** valvifer. Scales = 0.25 mm.

## Supplementary Material

XML Treatment for Dianous cyaneovirens

XML Treatment for Dianous haraldi

XML Treatment for Dianous huanghaoi

XML Treatment for Dianous yao
